# Vertical Zig-Zag Greenstick Osteotomy: A Novel Technique for Precise Nasal Bone Deformity Correction

**DOI:** 10.1055/a-2872-7788

**Published:** 2026-07-06

**Authors:** Elvin Alaskarov, Husam Vehbi, Aynur Aliyeva, Necdet Ozcelik

**Affiliations:** 1 Department of Otorhinolaryngology 218502 İstanbul Medipol University Health Care Practice and Research Center Esenler HospitalFatihİstanbulTurkey; 2 Department of Otorhinolaryngology 217747 Azerbaijan Medical UniversityBakuAzerbaijan; 3 Department of Radiology 218502 İstanbul Medipol University Health Care Practice and Research Center Esenler HospitalFatihIstanbulTurkey; 4 Department of Neuroscience 52998 Yeditepe UniversitesiIstanbulİstanbulTurkey; 5 Department of Surgery 47804 Denver Health and Hospital AuthorityDenverColoradoUnited States

**Keywords:** nasal bone deformity, osteotomy, rhinoplasty, nasal deviation, piezoelectric surgery, computed tomography analysis

## Abstract

**Background:**

Crooked nose deformities remain among the most challenging problems in rhinoplasty due to their combined aesthetic and functional implications. Conventional osteotomy techniques may lead to uncontrolled fractures or postoperative instability. This study aims to evaluate the effectiveness of the newly defined Vertical Zig-Zag Greenstick Osteotomy in correcting C-shaped and reverse C-shaped nasal deformities.

**Methods:**

A retrospective study was conducted on 37 patients (25 males, 12 females; mean age, 31.4 years) who underwent rhinoplasty with the Vertical Zig-Zag Greenstick Osteotomy between May 2020 and April 2023. Pre- and postoperative nasal deviation angles were measured using standardized photographs and high-resolution computed tomography (CT) scans at a minimum 12-month follow-up. Nasal bone thickness and length were assessed at three anatomical levels. The percentage of correction toward the ideal nasal axis (180 degrees) was calculated. Statistical comparisons were made using paired *t* -tests, with significance set at *p* < 0.05.

**Results:**

The mean preoperative deviation angle was 144 ± 5.47 degrees in C-shaped and 140 ± 2.64 degrees in reverse C-shaped deformities, improving postoperatively to 175 ± 3.24 degrees and 178 ± 2.20 degrees, respectively ( *p* < 0.001). The nasal bone on the deviated side was shorter (28.57 ± 1.34 mm) than the contralateral side (31.61 ± 1.41 mm; *p* < 0.001). Surgical outcomes were excellent in 73% and good in 24% of cases, with no complications or revisions.

**Conclusions:**

The Vertical Zig-Zag Greenstick Osteotomy provides a controlled, safe, and reproducible method for correcting crooked nose deformities, maintaining cortical continuity, and improving both aesthetic and functional results without additional osteoplasty.

## Introduction

Crooked nose deformities, often resulting from trauma, congenital asymmetry, or uneven growth, present one of the most challenging problems in rhinoplasty. These deformities cause both aesthetic distortion and functional airway obstruction, requiring precise surgical correction. 1 2

Crooked noses are typically categorized as C-shaped, reverse C-shaped, I-shaped, or S-shaped deformities, depending on the direction and extent of deviation. 3 4

Crooked nose deformities are characterized by asymmetric osseocartilaginous anatomy between the right and left nasal vaults. Differences in bone–cartilage configuration, angular orientation, and regional thickness are frequently observed between the two sides. Consequently, symmetrical surgical interventions applied equally to both nasal halves often fail to achieve optimal or durable correction. 5

Conventional lateral and medial osteotomy techniques may be insufficient in cases involving excessively irregular or laterally protruding (outwardly deviated) nasal bones. In such scenarios, surgeons commonly resort to multiple osteotomies and aggressive rasping or thinning of the deviated nasal bone to facilitate repositioning. 6 However, these maneuvers are largely based on the assumption that the primary pathological factor is excessive bone thickness rather than angular deformity. From a biomechanical perspective, preservation of the inner cortical bone during a greenstick osteotomy creates a controlled flexion axis, allowing gradual realignment of the nasal bone while maintaining structural stability. In contrast, complete osteotomies in thicker or irregular bony vaults may increase the risk of overmobilization, instability, and open-roof deformity.

This raises a critical question: Is the fundamental problem in outwardly deviated nasal bones related to increased bone thickness, or is it primarily an angular deformity caused by asymmetric bone length and orientation? If the deformity is predominantly angular, it remains unclear whether additional thinning or multiple complete osteotomies truly resolve the underlying biomechanical imbalance or merely introduce further instability.

In the present study, nasal bone thickness was systematically compared between the deviated and contralateral sides, and a novel Zig-Zag Greenstick Pattern Osteotomy was applied specifically to angularly deviated nasal bones.

## Methods

### Study Population

This retrospective study was conducted between May 2020 and April 2023 at a tertiary referral center. All surgeries were performed by a single experienced surgeon (E.A.). Ethical approval for this study was obtained from the Institutional Review Board (IRB; approval number: E-10840098-202.3.02-6439).

### Inclusion and Exclusion Criteria

Patients with C-shaped or reverse C-shaped nasal deformities requiring osteotomy for correction were included. Patients with a history of prior nasal surgery (including septoplasty, rhinoplasty, or endoscopic sinus surgery), uncontrolled systemic disease, major psychiatric disorders, age under 18 years, or a follow-up period of less than 1 year were excluded. These criteria were applied to minimize potential confounding factors that could influence surgical outcomes, including previous surgical trauma and systemic comorbidities.

All patients were informed about potential short-term and long-term risks, and written informed consent was obtained.

### Imaging Protocols

Preoperative and postoperative photographs were obtained from seven standardized angles: Frontal, lateral, oblique, bird's eye, and basal views. In addition, video recordings were archived. All photographs were captured using a Nikon camera equipped with a 70-mm macro lens.

Measurements of nasal deviation (axis) were performed using the open-source and free software ImageJ (Fiji, version 1.54i; National Institutes of Health, Bethesda, MD, United States). 7 The images were captured in the frontal view both preoperatively and at the 12-month postoperative mark.

The facial midline reference plane was first defined using the glabella (Point A) and the upper lip midpoint (Point D) to establish a standardized vertical facial axis. The nasion (Point B) and nasal tip (Point C) were then identified along this midline. The nasal deviation angle was calculated exclusively as the angle formed between lines B–E (from nasion to the point of maximal convexity) and E–C (from the convexity to the nasal tip), with Points A and D serving only as reference landmarks for midline alignment ( Fig. 1 ).

**Fig. 1:**
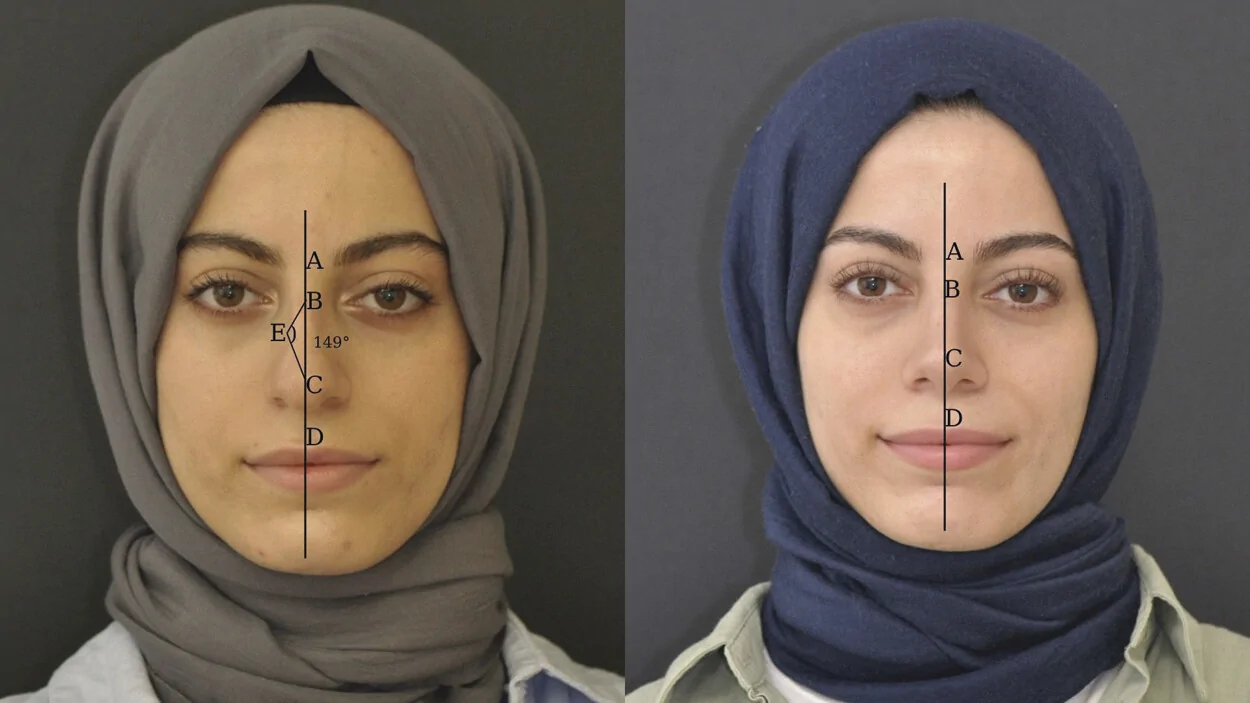
Measurement of nasal deviation angle in C-type crooked noses. Points A (glabella) and D (midpoint of the upper lip) define the facial midline reference axis. Points B (nasion), C (nasal tip), and E (point of maximal convexity) are used for angular calculation. The deviation angle is defined by the intersection of lines B–E and E–C.

Preoperative and postoperative angles (≥12 months) were measured using standardized imaging protocols. The ideal angular values were 180 degrees for C-type and reverse C-type deviations, representing a straight nasal dorsum.

Surgical outcomes were assessed by comparing postoperative angles to the ideal degree of correction, calculated as follows:

Ideal correction degree = Ideal angle − Preoperative angle

Achieved correction = Postoperative angle − Preoperative angle

Success rate = (Achieved correction/Ideal correction degree) × 100

According to this formula, a postoperative angle closer to the ideal angle indicates a higher success rate. The success of surgical correction was quantified by the percentage change toward the ideal angle (180 degrees), with outcomes classified as follows: Excellent (≥90% correction), Good (70–89%), Acceptable (50–69%), and Unsuccessful (<50%). This geometric classification was adapted from prior objective methods used in nasal deviation correction studies that emphasize angular deviation improvement as a reliable outcome parameter. 8 9

Preoperative high-resolution CT scans were used for analysis in all patients. The length of each nasal bone was measured on sagittal CT images, extending from the nasofrontal suture to the keystone area, where the bony nasal pyramid terminates.

The thickness of the bilateral nasal bones was measured on coronal CT sections at three standardized levels along the vertical axis of each nasal bone. For this purpose, the nasal bone was divided into three equal segments in the vertical plane: The upper third, middle third, and lower third ( Fig. 2 ). At each level, bone thickness was measured at the midpoint of the nasal bone on the corresponding coronal section, and the values were recorded for analysis. 10 11 12

**Fig. 2:**
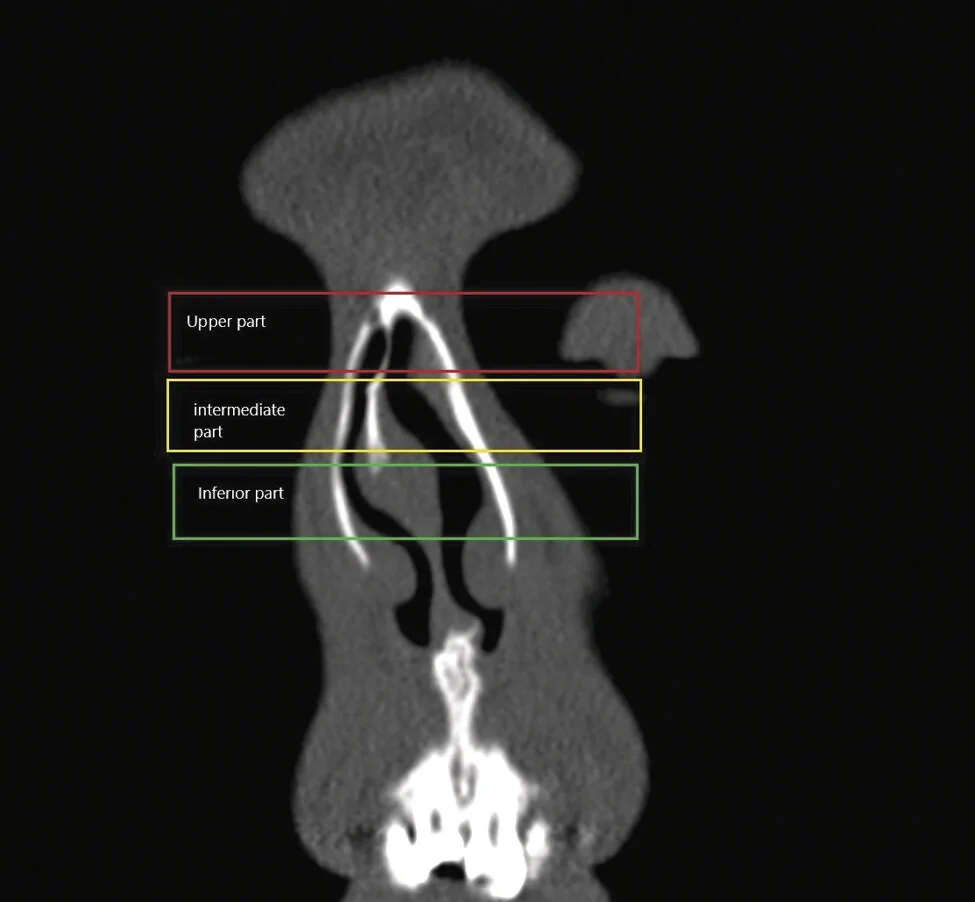
Preoperative coronal computed tomography images demonstrating nasal bone thickness measurements. The nasal bones were divided vertically into three equal segments—upper, middle, and inferior thirds—along the long axis of each nasal bone.

All measurements were performed by a single blinded observer who was unaware of the patients' clinical information and surgical outcomes.

Patients were followed for 12 to 24 months, and postoperative evaluations were conducted at least 12 months after surgery.

### Surgical Technique

All procedures were conducted under general anesthesia using an open-tip approach to ensure optimal exposure of the nasal framework. Supraperichondrial and subperiosteal dissection were meticulously performed to access both bony and cartilaginous structures. In cases requiring structural rhinoplasty, septoplasty was performed first, followed by hump resection when necessary.

The Vertical Zig-Zag Greenstick Osteotomy was performed using a piezoelectric device (Woodpecker, supplied by Selam Medical, Istanbul, Turkey) on the nasal bone deviated laterally from the midline in all patients included in the study. Because the osteotomy line was perpendicular to the long axis of the nasal bone, it was termed a Vertical Z Osteotomy. In patients with C-shaped crooked nose deformities, the osteotomy was performed vertically at the most deviated point of the right nasal bone, whereas in cases of reverse C-shaped deformities, the procedure was performed on the left nasal bone ( Fig. 3 ).

**Fig. 3:**
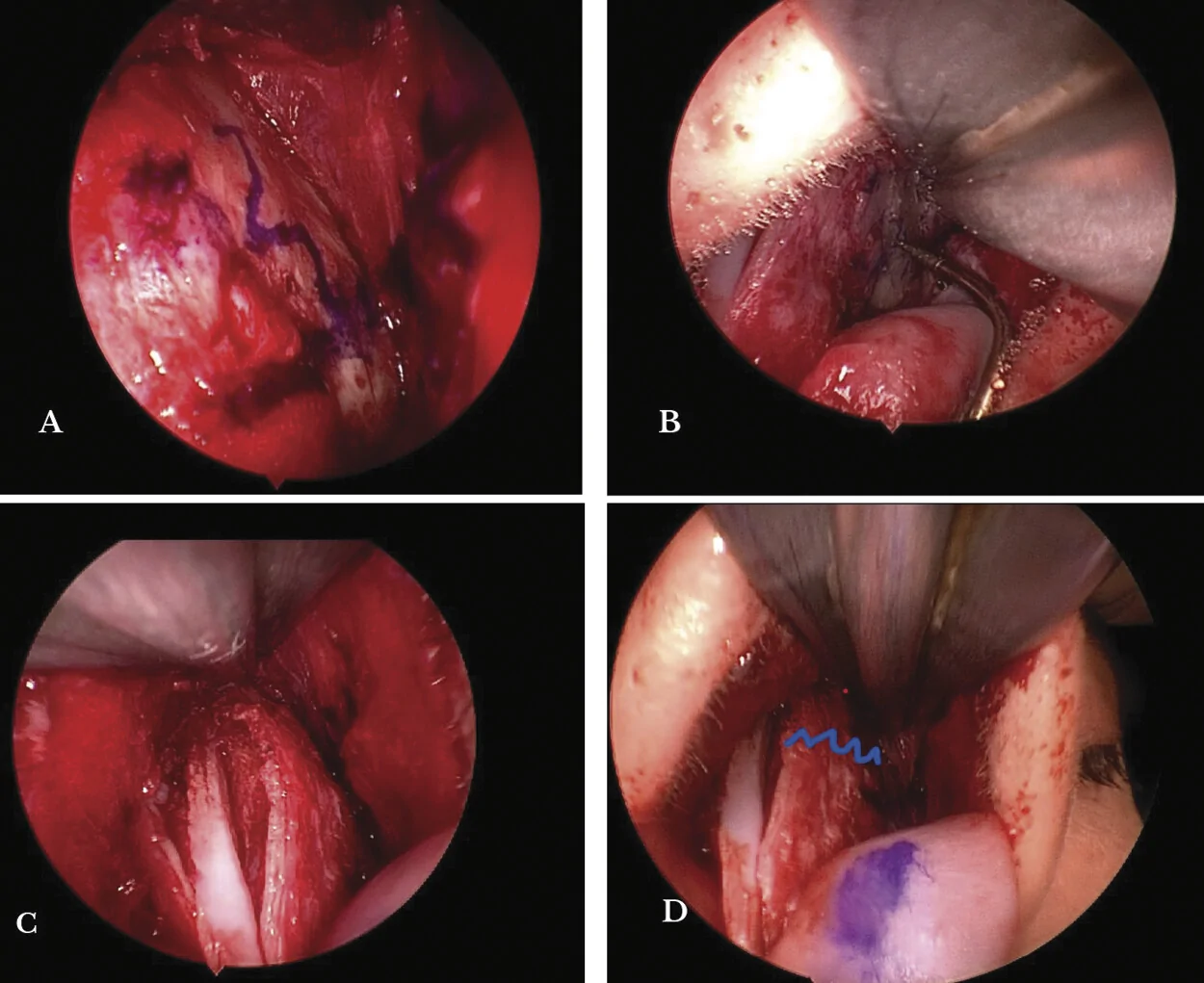
Intraoperative views illustrating the Vertical Zig-Zag Greenstick Osteotomy on the deviated nasal bone in a reverse C-shaped deformity. ( **A** ) The planned Vertical Zig-Zag Osteotomy line is marked on the left nasal bone at the point of maximal deviation. ( **B** ) Use of a piezoelectric device to achieve precise and controlled cortical cutting during the osteotomy procedure while preserving the medial hinge for greenstick mobilization. ( **C** ) Pre-osteotomy bird's-eye view demonstrating the outward deviation of the left nasal bone relative to the midline. ( **D** ) Post-osteotomy bird's-eye view showing medialization of the previously deviated nasal bone after controlled zig-zag greenstick bending. The blue line indicates the zig-zag osteotomy trajectory.

### Vertical Zig-Zag Greenstick Osteotomy

This technique involves selectively osteotomizing only the outwardly deviated portion of the nasal bone while preserving the inner cortical surface, thereby creating a controlled greenstick fracture. Zig-zag cuts are made along the laterally deviated nasal bone, starting from its outer surface and extending to the point where the deviation subsides. Gentle pressure is applied to correct the external deviation without compromising bone stability. The Z-shaped osteotomy line is then extended and connected to the inferior lateral osteotomy, leaving a bone segment intact to prevent structural collapse.

Osteotomy depth and the degree of medial cortical hinge preservation were intraoperatively adjusted according to the relative thickness and asymmetry of the nasal bones. In thicker bone segments, a more controlled and relatively shallower osteotomy was preferred to prevent complete cortical disruption and to maintain greenstick stability.

In preservation rhinoplasty cases utilizing the push-down technique, a vertical Zig-Zag Greenstick Osteotomy was performed on the deviated side, followed by additional osteotomies as necessary. A schematic representation of this technique is provided in Fig. 4 .

**Fig. 4:**
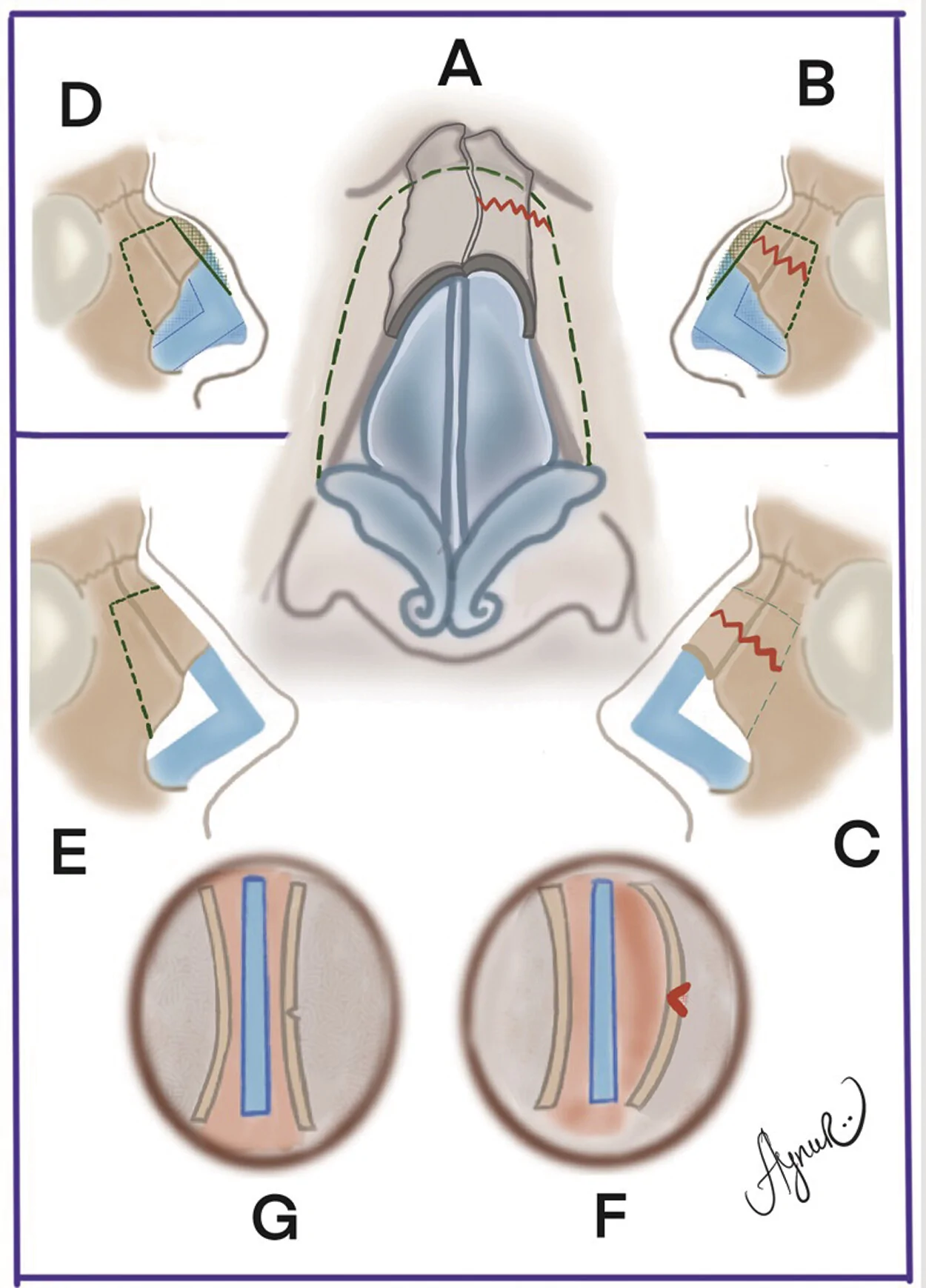
Schematic representation of the Vertical Zig-Zag Greenstick Osteotomy technique for correction of reverse C-shaped nasal deformity. ( **A** ) Preoperative anterior view demonstrating the planned zig-zag osteotomy trajectory on the externally deviated left nasal bone at the point of maximal convexity. ( **B** ) Preoperative lateral view illustrating the vertical orientation of the zig-zag osteotomy design and preservation of the medial cortical hinge to enable controlled greenstick mobilization. ( **C** ) Postoperative lateral view following zig-zag osteotomy, dorsal hump reduction, and transverse and lateral osteotomies on the deviated side. ( **D** ) Preoperative lateral view of the right (contralateral) nasal bone showing the planned transverse and double lateral osteotomy lines. ( **E** ) Postoperative lateral view of the contralateral nasal bone after dorsal hump reduction and completion of transverse and double lateral osteotomies. ( **F** ) Pre-osteotomy bird's-eye view demonstrating the nasal bone configuration and deviation relative to the midline septum. ( **G** ) Post-osteotomy bird's-eye view demonstrating medialization of the previously deviated left nasal bone after controlled greenstick bending and its connection with the inferior lateral osteotomy, resulting in correction of the external deviation. Red lines indicate the zig-zag osteotomy trajectories and auxiliary osteotomy lines. Blue lines represent the nasal midline/septum. The illustration was created specifically for this manuscript by Aynur Aliyeva, MD, PhD(c). Written permission for publication has been obtained from the illustrator.

A single Z-osteotomy was performed on the lateral wall of the nasal bone at the point of maximal deviation in each patient ( *n* = 37), with each arm of the Z measuring approximately 5 to 7 mm in length and angled at 45 to 60 degrees.

### Osteotomy Approach Based on Nasal Deviation

• For the deviated (short) nasal bone:
  ▪ Vertical zig-zag greenstick osteotomy
  ▪ Single low-to-low osteotomy
  ▪ Transverse osteotomy
  ▪ Medial-oblique osteotomy
• For the contralateral (long) nasal bone:
  ▪ Double lateral osteotomy
  ▪ Transverse osteotomy

This approach ensured effective correction without the need for additional osteoplasty following the Vertical Zig-Zag Greenstick Osteotomy. The procedure successfully corrected the external deviation of the nasal bone while simultaneously increasing the length of the shorter nasal bone and improving the parallel alignment of its cranial and caudal edges.

### Bone Stability and Risk Mitigation

A potential drawback of the technique is the risk of nasal bone collapse, which can be minimized by utilizing piezoelectric or micro-saw tools for precise osteotomy control. Additionally, preserving an intact bone segment between the inferior lateral osteotomy lines significantly reduces the likelihood of collapse. In this case series, two patients required long spreader grafts for additional structural support.

### Septoplasty and Additional Grafts

Septal correction and adjunctive grafting procedures were performed in a stepwise manner based on intraoperative structural requirements, with the specific techniques and graft types selected to optimize midline alignment, dorsal stability, and functional support. In cases requiring septal correction, the following techniques were applied:

- Septal scoring was performed in 25 patients to facilitate realignment.
- The swinging-door technique was used in eight patients to reposition the septum to the midline.
- Total septal reconstruction was required in four patients, involving fixation of the L-strut cartilage to the nasal spine at the keystone area.
- For structural reinforcement, two long spreader grafts and bilateral spreader flaps were used in 28 patients.
- Double spreader grafts were placed on the deviated side in three patients, while bilateral spreader grafts were positioned in six patients to optimize nasal alignment and stability.

This comprehensive approach ensured structural integrity, optimized functional correction, and contributed to favorable long-term aesthetic outcomes.

### Statistical Analysis

These comparisons were performed using statistical tests to assess the significance of preoperative and postoperative measurements. Paired *t* -tests were conducted to find variations in nasal angle and stability of bones, and if the *p* -value obtained was <0.05, then it was considered statistically significant. The confidence intervals were also calculated for the parameters to ensure the reliability of the observed changes after surgery. A post hoc power analysis was conducted using the effect sizes derived from the changes in nasal deviation angles. For the C-type group ( *n* = 15), the improvement from 144 ± 5.47 degrees to 175 ± 3.24 degrees yielded a Cohen's *d* of 6.90. For the reverse C-type group ( *n* = 22), the change from 140 ± 2.64 degrees to 178 ± 2.20 degrees resulted in a Cohen's *d* of 15.64. Using these values, the statistical power for both groups was calculated to be 1.00 at α = 0.05, indicating sufficient power to detect significant improvements in nasal alignment.

## Results

### Patient and Surgery Data

A total of 37 patients (mean age: 31.4 years; age range: 18–44 years) underwent rhinoplasty to correct C-type or reverse C-type crooked nose deformities. Among these patients, 25 (67%) were male, and 12 (33%) were female. Regarding the type of deformity, 15 patients (40%) had a C-shaped crooked nose, while 22 patients (60%) had a reverse C-type deformity.

Surgical techniques varied among patients. Structural rhinoplasty with hump resection was performed on 29 patients, while 8 patients underwent preservation rhinoplasty with a bony cap push-down technique.

Osteotomy techniques were selected based on the specific nasal deviation. On the deviated side, the following osteotomies were performed: Vertical zig-zag greenstick fracture, single low-to-low osteotomy, transverse osteotomy, and medial-oblique osteotomy. On the contralateral side, double lateral and transverse osteotomies were applied ( Table 1 ).

**Table 1:** Demographic and surgical data of patients

Characteristics	Number of patients, *n* = 37
Age, mean years (min–max)	31.4 (18–44)
Males, *n* (%) Females, *n* (%)	25 (67%) 12 (33%)
Type of crooked nose, *n* (%)	C-shaped deformity, 15 (40%) Reverse C type, *n* = 22 (60%)
Surgery type, *n*	Structural hump resection, *n* = 29 Preservation of the bony cap (push-down technique), *n* = 8

Values are presented as number (percentage).

### Preoperative and Postoperative Measurements

Preoperative and postoperative angle measurements were analyzed based on the type of nasal deformity ( Table 2 ). In patients with C-shaped deformities ( *n* = 15), the mean preoperative angle was 144 ± 5.47 degrees, which significantly improved to 175 ± 3.24 degrees at the 12-month postoperative evaluation ( *p* < 0.001). Similarly, in patients with reverse C-shaped deformities ( *n* = 22), the mean preoperative angle was 140 ± 2.64 degrees, which increased to 178 ± 2.20 degrees postoperatively ( *p* < 0.001). These findings indicate a statistically significant improvement in nasal alignment following surgery, regardless of the type of deformity.

**Table 2:** Comparison of preoperative and postoperative angle measurements according to deformity type

	Preoperative angle ± SD	Postoperative angle ± SD	*p* -Value a
C-shaped deformity	144 ± 5.47	175 ± 3.24	<0.001
Reverse C-shaped deformity	140 ± 2.64	178 ± 2.20	<0.001

Abbreviation: SD, standard deviation.

The ideal nasal dorsum alignment is defined as 180 degrees.

a Paired samples *t* -test.

Preoperative CT scans were used to measure the thickness and length of the nasal bone in patients with an asymmetric bony vault ( Table 3 ). Bone thickness measurements indicated no significant differences between the deviated and contralateral sides in most regions of the upper and intermediate parts. However, in the intermediate part, the middle and high levels demonstrated statistically significant differences, with the deviated side being slightly thicker than the contralateral side ( *p* = 0.042 and *p* = 0.038, respectively). Significant differences were noted in the inferior part at the middle and high levels, where the deviated side exhibited greater thickness than the contralateral side ( *p* < 0.001 for both).

**Table 3:** Comparison of bone thickness and length between the deviated side and the contralateral side in asymmetric bony vaults ( *n* = 37)

Nose bone measurement (mm) ± SD	Deviated side	Contralateral side	*p* -Value a
Upper part			
Low	2.15 ± 0.16	2.08 ± 0.21	0.473
Middle	2.10 ± 0.12	1.98 ± 0.16	0.461
High	2.20 ± 0.20	2.14 ± 0.18	0.586
Intermediate part			
Low	1.77 ± 0.24	1.73 ± 0.17	0.637
Middle	1.73 ± 0.32	1.68 ± 0.26	0.042
High	1.82 ± 0.25	1.77 ± 0.18	0.038
Inferior part			
Low	2.54 ± 0.46	2.41 ± 0.44	0.386
Middle	2.64 ± 0.53	2.35 ± 0.36	<0.001 a
High	2.68 ± 0.51	2.30 ± 0.42	<0.001 a
Nasal bone length	28.57 ± 1.34	31.61 ± 1.41	<0.001 a

Abbreviation: SD, standard deviation.

*p* < 0.05 was considered statistically significant.

a Paired samples *t* -test.

Regarding nasal bone length, the contralateral side was significantly longer than the deviated side (28.57 ± 1.34 mm vs. 31.61 ± 1.41 mm, *p* < 0.001).

Surgical success rates were assessed according to the type of deformity ( Table 4 ). About 73% of patients ( *n* = 27) achieved excellent outcomes, while 24% ( *n* = 9) achieved good results. No cases were classified as unsuccessful.

**Table 4:** Surgical success rates by deformity type

Surgical success	C type, *n* = 15	Reverse C type, *n* = 22	Total, *n* = 37
Excellent	11 (73%)	16 (73%)	27 (73%)
Good	3 (20%)	6 (27%)	9 (24%)
Acceptable	1 (7%)	0	1 (3%)
Unsuccessful	0	0	

Among patients with C-type deformity ( *n* = 15), 73% ( *n* = 11) achieved excellent outcomes, while 20% ( *n* = 3) had good outcomes. One patient (7%) was classified as having an acceptable result. In patients with reverse C-type deformity ( *n* = 22), 73% ( *n* = 16) experienced excellent outcomes, and 27% ( *n* = 6) had good outcomes. Notably, no complications or revision surgeries were reported during the follow-up period.

Figure 5A, B compares the preoperative and postoperative frontal views of a patient with a C-shaped crooked nose. Figure 6A, B illustrates the preoperative and postoperative frontal views of another patient presenting with a reverse C-shaped nasal deformity.

**Fig. 5:**
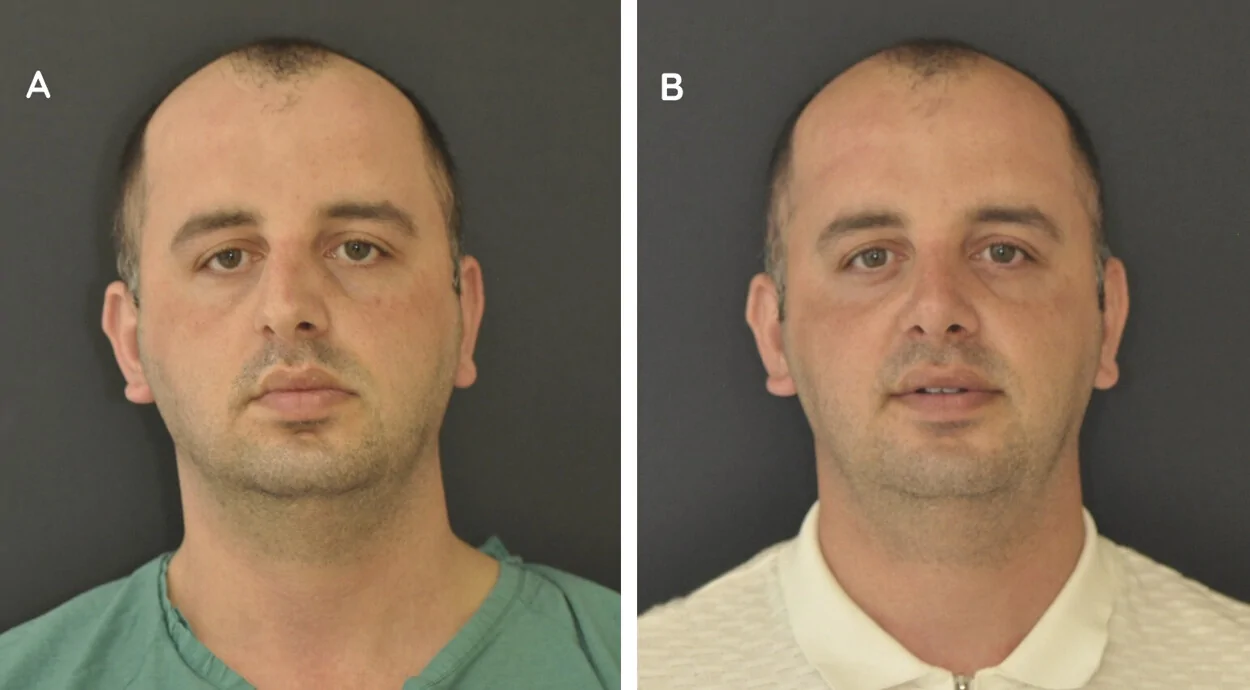
Preoperative ( **A** ) and 12-month postoperative ( **B)** frontal views of a patient with a C-type crooked nose.

**Fig. 6:**
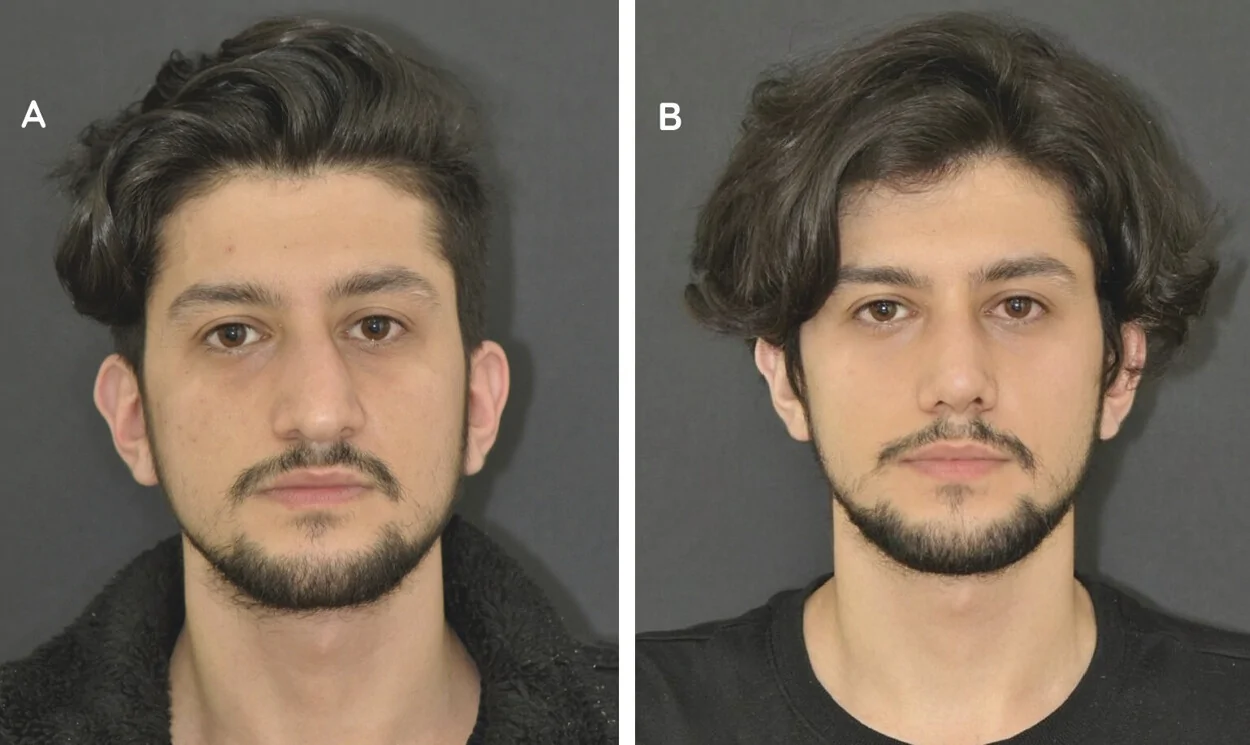
Preoperative ( **A** ) and 12-month postoperative ( **B** ) frontal views of a patient with a reversed C-type crooked nose.

No complications or revision surgeries were reported during the follow-up period.

## Discussion

Achieving durable correction of crooked nose deformities remains one of the most complex challenges in rhinoplasty, primarily because of asymmetric nasal bone length, orientation, and biomechanical behavior during osteotomy. Conventional lateral and medial osteotomies primarily aim to reposition the nasal bones by creating complete fractures; however, in markedly asymmetric bony vaults, this approach may result in uncontrolled fracture lines, instability, or the need for excessive bone thinning and secondary osteoplasty. 13

Rather than representing a modification of conventional osteotomy execution, the zig-zag greenstick osteotomy is conceptually designed to correct angular deformity by creating a controlled flexion axis while preserving cortical continuity. This biomechanical principle distinguishes the technique from complete fracture-based osteotomies and underlies its stability in asymmetric bony vaults.

The fundamental conceptual distinction of the Vertical Z Greenstick Osteotomy lies in addressing *angular deviation rather than bone volume* . Classical teaching often attributes lateral nasal bone deviation to excessive bone thickness, leading surgeons to perform aggressive rasping, burring, or multiple osteotomies on the convex side. However, our tomographic findings demonstrate that the primary deformity in crooked noses is not generalized bone hypertrophy but asymmetric bone length and angular malalignment ( Table 3 ).

In the present study, the deviated nasal bone was significantly shorter than the contralateral side, while thickness differences were limited and region-specific. These findings challenge the rationale of routine bone thinning and support the hypothesis that angular correction through controlled mobilization is biomechanically more appropriate than volume reduction. The Vertical Z Greenstick Osteotomy was, therefore, designed to selectively weaken the convex portion of the nasal bone, allowing controlled medialization while preserving cortical continuity.

From a biomechanical standpoint, excessive bony deformity in crooked noses may predispose to uncontrolled fractures during osteotomy, particularly when complete fractures are attempted in rigid or asymmetric bony vaults. In such cases, the use of advanced surgical instruments, such as piezoelectric devices or micro-saws, enhances precision and fracture control. 14 15 In our series, all osteotomies were performed using piezoelectric instrumentation. The Vertical Zig-Zag osteotomy was applied as a greenstick fracture pattern, with deliberate preservation of an intact bony segment between the inferior lateral osteotomy lines to reduce the risk of nasal bone collapse. Additional medial support with long spreader grafts was required in two patients, highlighting the importance of selective structural reinforcement in specific cases. Although no postoperative airway narrowing at the high bony septum was observed, this remains a theoretical concern in anatomically narrow nasal vaults and should be considered during surgical planning.

A prospective study analyzing the bony vault anatomy of rhinoplasty patients using CT scans reported that the bony vault thickness was significantly thinner in females compared to males. Additionally, in asymmetric bony vaults, the deviated side was thicker than the contralateral side along all osteotomy paths. 16 Our case series used CT scans to measure the nasal bone thickness on both the deviated and contralateral sides. No significant difference in thickness was observed between the upper and intermediate segments of both nasal bones. This finding further supports the concept that aggressive thinning of the convex nasal bone may be unnecessary and potentially detrimental.

Previous studies have demonstrated that nasal bone length varies among different ethnic groups. In one study, the average nasal bone length in Korean males was 2.59 ± 0.38 cm, while in females, it was 2.45 ± 0.37 cm. 17 A study conducted in Turkey reported nasal bone lengths of 2.4 ± 0.2 cm in males and 2.3 ± 0.2 cm in females. 18

Our study observed a significant difference in nasal bone length between the right and left nasal bones. The average length of the deviated side was 2.8 cm, while the contralateral side measured 3.1 cm. This discrepancy, when compared to the existing literature, can be attributed to the fact that all patients in our case series presented with crooked noses.

From a practical standpoint, nasal bone thickness played a role in guiding osteotomy depth and hinge preservation rather than determining a fixed surgical threshold. Instead of applying strict numerical criteria, the technique allows intraoperative adaptation based on relative asymmetry and bone resistance, which may contribute to more controlled and stable outcomes compared to conventional complete osteotomies.

It is important to emphasize that the Vertical Zig-Zag Greenstick Osteotomy is not intended as a universal standalone solution. In cases with extremely thick, rigid, or multiply deformed bony vaults, greenstick correction alone may be insufficient to overcome residual resistance. In such scenarios, adjunctive procedures—such as transverse, medial-oblique, or contralateral double lateral osteotomies—are necessary to achieve stable and symmetric alignment. Clearly defining these boundaries enhances the reproducibility and safety of the technique.

In our study, no significant difference in bone thickness was observed between the upper and intermediate regions of both nasal halves. Therefore, performing burring or contouring on the deviated side of the deviated segment may excessively thin the bone, which could compromise structural integrity. This finding underscores the importance of the Vertical Zig-Zag Greenstick Osteotomy, as it enables correction of the laterally deviated segment (convexity) without the need for bone thinning.

### Limitations

The main limitations of this study include its retrospective design, limited sample size, and absence of a control group using conventional osteotomy methods. Additionally, the follow-up period (up to 24 months) may not fully capture long-term stability or relapse. Functional outcomes such as nasal airflow and resistance were not objectively measured and should be incorporated into future studies.

In patients with crooked nose deformity, significant internal and external nasal valve dysfunction is frequently present and represents a major contributor to preoperative nasal obstruction. Previous studies have demonstrated that correction of these valve pathologies during rhinoplasty is associated with substantial postoperative improvement in nasal airflow and breathing function. 19

All angular measurements were performed by a single observer, and inter- and intraobserver reliability analyses were not conducted. This may introduce potential measurement bias and should be considered when interpreting the results.

In our case series, no postoperative complaints of nasal obstruction were observed during follow-up. However, it would not be scientifically appropriate to attribute this functional improvement specifically to the zig-zag greenstick osteotomy technique. As noted above, restoration of nasal valve competence alone is generally sufficient to achieve marked airway improvement in this patient population.

Despite these limitations, the technique demonstrated a high success rate with no complications or revision surgeries.

### Conclusion

The Vertical Zig-Zag Greenstick Osteotomy provides a precise and reproducible technique for the correction of C-shaped and reverse C-shaped nasal bone deformities. By preserving cortical integrity and reducing the risk of uncontrolled fractures, this method offers stable bone alignment and favorable aesthetic outcomes without the need for additional osteoplasty. The technique represents a reliable alternative to conventional osteotomies, particularly in cases of pronounced bony asymmetry.

Future prospective multicenter studies with larger sample sizes and objective functional assessments are recommended to validate the long-term stability and clinical applicability of this approach.
